# Oxidative Stress and the Intersection of Oncogenic Signaling and Metabolism in Squamous Cell Carcinomas

**DOI:** 10.3390/cells10030606

**Published:** 2021-03-09

**Authors:** Joshua H. Choe, Simbarashe Mazambani, Tae Hoon Kim, Jung-whan Kim

**Affiliations:** 1 Department of Biological Sciences, Columbia University, New York, NY 10027, USA; 2 Department of Biological Sciences, The University of Texas at Dallas, Richardson, TX 75080, USA; sxm170074@utdallas.edu (S.M.); genome@utdallas.edu (T.H.K.); 3 Research and Development, VeraVerse Inc., 50-1 Yonsei-ro, Seodaemun-gu, Seoul 03722, Korea

**Keywords:** squamous cell carcinoma, metabolism, oxidative stress

## Abstract

Squamous cell carcinomas (SCCs) arise from both stratified squamous and non-squamous epithelium of diverse anatomical sites and collectively represent one of the most frequent solid tumors, accounting for more than one million cancer deaths annually. Despite this prevalence, SCC patients have not fully benefited from recent advances in molecularly targeted therapy or immunotherapy. Rather, decades old platinum-based or radiation regimens retaining limited specificity to the unique characteristics of SCC remain first-line treatment options. Historically, a lack of a consolidated perspective on genetic aberrations driving oncogenic transformation and other such factors essential for SCC pathogenesis and intrinsic confounding cellular heterogeneity in SCC have contributed to a critical dearth in effective and specific therapies. However, emerging evidence characterizing the distinct genomic, epigenetic, and metabolic landscapes of SCC may be elucidating unifying features in a seemingly heterogeneous disease. In this review, by describing distinct metabolic alterations and genetic drivers of SCC revealed by recent studies, we aim to establish a conceptual framework for a previously unappreciated network of oncogenic signaling, redox perturbation, and metabolic reprogramming that may reveal targetable vulnerabilities at their intersection.

## 1. Introduction

Squamous epithelia are comprised of proliferative basal layers and increasingly differentiated suprabasal layers in the outermost layer of the skin, inner mouth, esophagus, and anogenital regions and may also develop from squamous metaplasia, a replacement of non-squamous epithelium by squamous epithelium, induced by injury or other stress conditions in the respiratory and urinary tracts [1,2]. SCCs can arise from both stratified squamous epithelium and non-squamous epithelium across many anatomical sites including most frequently in the skin, lung, head and neck, esophagus, and cervix [2]. In addition, SCCs may develop from non-squamous epithelial tissue through squamous transdifferentiation [2,3,4,5]. Despite the diverse locations in which they are found, SCCs commonly originate from proliferative basal cells. In contrast, other cell types of squamous epithelia, such as transit-amplifying progenitors and terminally differentiated, non-proliferative epithelial cells, are unable to generate malignant tumors even with the introduction of oncogenic mutations [6,7,8].

Recent comprehensive genomic characterizations of common SCCs demonstrate similar mutational landscapes even across different SCCs including aberrations in *TP53*, *TP63*, *SOX2*, *PIK3CA*, *KEAP1*, and *NFE2L2* genes [9,10,11,12,13,14,15]. However, targeted therapies remain limited, and approved RTK inhibitors such as EGFR inhibitor cetuximab only modestly improve survival in SCC patients and inevitably lead to clinical resistance [2,3,4,5,16,17,18]. Although with current standards of treatment including surgical resection and radiation of low-risk or early stage SCCs are highly curable (~95–98%), locally advanced and metastatic SCCs retain poor five-year survival rates. These advanced cancers remain difficult to treat, and only highly toxic chemotherapy regimens, such as 5-fluorouracil and platinum-based agents, are available for these cases. As a result of high cellular heterogeneity and their intrinsic ability to enter into drug-resistant TGF-beta-mediated quiescent phases, SCCs are also difficult to treat with monotherapeutic approaches and frequently display chemoresistance [17,19]. A preponderance of recent evidence characterizing an SCC-specific convergence of oncogenic signaling and metabolism may suggest a novel paradigm in the treatment of advanced SCC: synergistically targeting the nexus of oncogenic and metabolic pathways [16,18,20].

Reactive oxygen species (ROS) come in many flavors, among which superoxide, hydrogen peroxide, and hydroxyl radicals are most well studied in the context of cancer, and can be produced from both endogenous and exogenous sources [21]. ROS play a complex and as yet incompletely understood role in cancer, playing both tumor promoting and tumor suppressive roles depending on ROS levels and stage of tumor progression [22]. At moderate levels, ROS may promote tumor formation, increased proliferation and survival signaling, genomic instability, and increased motility. Excessive ROS, however, induces arrest, senescence, and death in cancers. Specifically, some types of ROS can drive DNA damage in normal tissues, which can result in the first mutations that can set a cell on the path to tumor formation [23]. Further, ROS can activate pro-cancer signaling pathways including p38 mediated MAP kinase activation and hypoxia inducible factor (HIF) stabilization [24,25,26]. Conversely, when left unchecked, ROS levels can cause cell death through a variety of mechanisms that may be exploited to treat cancer [27]. However, tumor metabolic reprogramming can help to protect cancers from ROS toxicity by driving the production of the cell’s primary antioxidant glutathione (GSH), and the NADPH required to recycle oxidized GSH back to its reduced form [28]. Recently, analysis into the composition of cutaneous SCCs (CSCC) revealed distinct subpopulations within tumors that recapitulate normal keratinocyte populations, basal, cycling, and differentiated, but have acquired novel oncogenic characteristics including the upregulation of glycolysis and response to ROS and a downregulation of apoptosis [29]. Although the specific contributions of various ROS species and their regulation in different cellular compartments are undoubtedly relevant in cancer, here we focus on ROS as damaging agents that necessitate cellular adaptation in SCCs.

In this review specifically, we discuss commonalities in etiological factors that point toward oxidative stress as a key driver of SCC pathogenesis and posit that despite the disparate origins of SCCs, oxidative stress plays a significant role in driving the acquisition of metabolic and oncogenic signatures unique to SCCs.

## 2. Commonalities in Squamous Etiology and Mechanisms of Carcinogenesis

Epidemiological studies have long demonstrated a strong association between SCCs and a myriad of environmental risk factors including UV exposure, smoking, alcohol consumption, and human papilloma virus (HPV) infection, among others (Table 1) [30,31,32]. Despite incredible diversity in risk factors causally associated with SCCs, key mechanistic similarities in major risk factors driving squamous-specific tumorigenesis exist even across SCCs of different anatomical origin [2]. Specifically, induction of ROS is a feature common amongst the myriad SCC risk factors. Thus, oxidative stress may be one of the key contributors in the oncogenesis of SCCs in conjunction with other mutagenic events that ultimately drive the acquisition of antioxidant defense strategies.

### 2.1. Cigarette Smoking

Cigarette smoking is an irrefutably critical risk factor for cancer and has been traditionally more associated with lung SCC (LSCC) development. Furthermore, among the 12 cancers associated with cigarette smoking, SCCs generally rank highest in cancer deaths attributable to such [33,34,35,36,37]. A veritable cocktail of carcinogenic compounds, cigarette smoke induces tumorigenesis primarily via DNA adduct formation and G→T transversions, especially in the tumor suppressor gene *TP53* [38,39,40,41]. Additionally, nitric oxide and quinone-hydroquinone complexes in cigarette smoke not only precipitate oxidative DNA damage, as indicated by several studies revealing increased 8-hydroxy-2′-deoxyguanosine (8-OHdG) in lung tissue and elevated lipid peroxidation and nitric oxide products in head and neck SCC (HNSCC) patients, but also provides selective pressure for cells able to upregulate antioxidant programs [41,42,43,44,45,46]. Furthermore, dietary antioxidants demonstrate a protective function against LSCCs especially even in heavy smokers [47].

### 2.2. Alcohol

Alcohol consumption has been most closely associated with HNSCCs and esophageal SCCs (ESCCs) among all cancer types, with some limited causal links with lung cancers and CSCC, with genomic analysis identifying alcohol drinking-related mutational signatures in ESCCs [48,49,50,51]. An incredible diversity in mechanisms of alcohol-induced carcinogenesis has been proposed including through the genotoxic effects of acetaldehyde via DNA adduct formation, inflammation, alterations in folate and retinoid metabolism, and oxidative stress [52,53,54]. Alcohol precipitates oxidative stress via free radical formation during cytochrome p450 isoenzyme-mediated ethanol metabolism, increasing levels of free iron within a cell, formation of the alcohol-derived 1-hydroxyethyl radical, and by depleting levels of key antioxidants such as GSH [55]. Significantly, a recent meta-analysis of alcohol consumption and cancer risk demonstrates the highest relative risk of ESCC development whereas the relative risk of esophageal adenocarcinoma (ADC) remains nearly unchanged, suggesting that oncogenic insults implicating ROS may preferentially drive SCC development [56].

### 2.3. Human Papilloma Virus

HPV types 16 and 18 have long been causally associated with cervical and oropharyngeal SCCs, significantly more prevalent than corresponding ADC subtypes, with evidence also linking HPV infection to cutaneous and esophageal development [57,58,59,60,61]. Classically, HPV oncoproteins E6 and E7 are thought to drive carcinogenesis primarily by binding and inactivating tumor suppressors p53 and Rb, respectively, thereby inducing aberrant proliferation and genomic instability. These classical oncogenic viruses also implicate metabolic alterations in SCC. NADPH oxidase (NOX) family genes *DUOX1*, *DUOX2*, and *NOX2*, which catalyze the production of superoxide free radicals, were found to be significantly higher expressed in cervical SCC (CESCC) tissues than cervical ADC and significantly increased in HPV16-infected patients [62]. Furthermore, HPV oncoproteins E6 and E7 activate NOX2 in HNSCCs, and both HPV E6, and its shorter isoform E6* has been revealed to attenuate the expression of antioxidants superoxide dismutase isoform 2 (SOD2) and glutathione peroxidase (GPx) [63,64].

### 2.4. UV

UVA and UVB radiation are definite carcinogens as demonstrated both epidemiologically and experimentally, and are closely associated with increased incidences of basal cell carcinoma (BCC) and SCC [65]. UVA and UVB radiation drive transformation through disparate mechanisms. The higher energy UVB radiation (280–315 nm), considered more tumorigenic and direct, induces covalent linkages between adjacent pyrimidine bases to form either cyclobutane pyrimidine dimers (CPD) or 6-4 pyrimidine pyrimidone photoproducts, typically resulting in C→T substitutions [66]. On the other hand, UVA radiation (315–400 nm) comprises the majority of the UV spectrum reaching the Earth’s surface and is mutagenic through indirect mechanisms involving generation of ROS via absorption by photosensitizers such as porphyrins and NADH [67]. The subsequently generated singlet oxygen reacts with guanine to form 8-oxo-2’-deoxyguanosine, resulting in G→T transversions [67]. Furthermore, UVA may promote carcinogenesis through non-DNA damage mechanisms by long-term ROS and reactive nitrogen species generation, inflammation, genomic instability, and immune suppression [68,69,70,71].

Despite the vastly disparate mechanisms by which etiological risk factors for SCCs exert pro-oncogenic effects, a common theme implicated in the pathophysiology of SCCs, even across major risk factors and SCCs of distinct anatomical origins, emerges. Oxidative stress may be the key-differentiating factor driving SCC development preferentially in the context of critical pro-oncogenic events such as the loss of p53 function over other cancer types. Given the protective barrier and selective transport functions of stratified epithelia from which SCCs originate, such cells remain highly exposed to environmental stresses and may undergo squamous metaplasia as an adaptive mechanism in response to chronic injury [72]. A survey of normal tissues showed the highest rates of somatic clonal expansion in the lung, skin, and esophagus, suggesting that exposure to environmental factors underlie mutational events in these tissues prone to SCC [73]. The finding that increased ROS modulates ADC to SCC transdifferentiation suggests that SCC transcriptional programs may more adequately respond and proliferate under oxidative stress conditions [74]. Thus, we can reasonably speculate that cancers that originate from squamous epithelia may not only be more poised to oncogenically transform but also co-opt squamous-lineage specific programs intended to mitigate stress to ultimately promote survival.

## 3. Metabolic Dependencies in Squamous Cell Carcinomas

### 3.1. Glucose

Tumors engage in metabolic reprogramming to fulfill cellular bioenergetic and anabolic needs as well as to fuel antioxidant production and generate redox cofactors. Emerging evidence has identified distinct metabolic phenotypes characteristic of SCCs. Diverting glycolytic flux into antioxidant-generating pathways, such as the pentose phosphate pathway (PPP) and de novo serine biosynthesis, remain critical strategies cancers utilize to combat toxic oxidative stress and may thereby metabolically reprogram to upregulate glycolysis and glucose influx [75]. Goodwin et al. demonstrate that LSCCs are uniquely reliant on glucose uptake mediated by significantly elevated glucose transporter 1 (GLUT1) with high GLUT1-expressing SCC patients exhibiting decreased survival [76]. This addiction to glucose renders LSCCs exquisitely vulnerable to glycolytic inhibition in vitro and in vivo while glucose remains dispensable for lung ADCs. Furthermore, multiple studies have identified higher F18-fluorodeoxyglucose (^18^F-FDG) uptake via ^18^F-FDG PET imaging in patients with LSCC tumors as compared to ADC tumors as well as higher risk of risk of recurrence and lower survival in tumors with high uptake of ^18^F-FDG [76,77,78,79,80].

**Figure 1:**
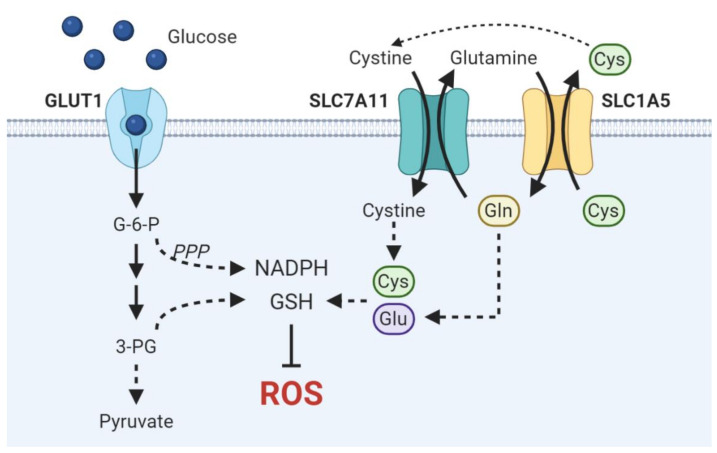
Schematic overview of metabolic pathways critical in driving antioxidant production in SCCs. Enhanced glucose uptake fuels NADPH and GSH synthesis via the pentose phosphate pathway and de novo serine biosynthesis, respectively. Cysteine and glutamate are necessary to synthesize the GSH precursor γ-glutamylcysteine. SLC7A11 and SLC1A5 are functionally coupled to drive cystine import.

This distinct metabolic feature, rather than a characteristic isolated to LSCCs specifically, is a convergent phenotype intrinsically associated with SCCs as a whole. Hsieh et al. reveal that head and neck, lung, esophageal, and cervical SCCs are the highest GLUT1-expressing cancers and critically rely on GLUT1-mediated elevated glucose influx to fuel antioxidant production, ultimately indicating a squamous lineage-specific convergent phenotype [81]. The essential contribution of GLUT1 overexpression to antioxidant production is demonstrated by the clear association of GLUT1 overexpression with resistance to radiation therapy, which primarily exerts cytotoxic effects via direct genomic damage and indirect free radical generation, in oral, cervical, and head and neck SCCs [82,83,84]. By redirecting enhanced glucose flux driven by GLUT1 overexpression into the PPP, a major cellular source of the NADPH used to recycle oxidized GSH, and the de novo serine biosynthesis pathway, which can provide the glycine component of glutathione and mitochondrial NADPH, SCCs generate elevated NADPH and GSH to ultimately maintain redox homeostasis and heighten antioxidant defenses (Figure 1) [81,85]. Inhibiting GSH metabolism via buthionine sulfoxamine treatment attenuated ESCC progression in vivo and enhanced cell killing in conjunction with thioredoxin inhibition in HNSCC cell lines [86,87]. Inhibition of the PPP via glucose-6-phosphate dehydrogenase (G6PD) blockade induced cell death in HNSCC cell lines by precipitating ER stress and accumulation of ROS [88]. Moreover, integrative transcriptomic and metabolomic analyses in vivo of lung ADC and SCC tumors reveal a distinctive metabolic signature of SCCs characterized by enhanced glucose catabolism into glycolysis, Krebs cycle, and pentose phosphate, GSH, and serine biosynthesis [89].

HPV-positive HNSCCs may be the exception to this metabolic commonality. HPV oncoproteins E6 and E7 have traditionally been thought to increase glucose uptake and glycolysis by inhibiting p53, interacting with c-Myc, binding PKM2, and enhancing both HIF1 and HK2 expression, among other mechanisms [90,91,92,93]. However, recent evidence demonstrates that HPV-positive HNSCCs exhibit comparatively decreased glycolysis and lower expression levels of genes involved in glycolysis [94,95,96]. Although the conflicting functions of HPV in carbohydrate metabolism warrants further investigation, emerging evidence suggests that HPV-associated cancers may need to be considered as metabolically distinct from their counterparts.

### 3.2. Glutamine

While redox homeostasis is accomplished primarily through glycolytic upregulation in SCC-specific metabolic programs, SCCs may also exploit amino acid transport to drive antioxidant anabolism and impart metabolic flexibility under glucose limiting conditions. Flores et al. suggest that lactate dehydrogenase A (LDHA) and aerobic glycolysis may be dispensable in CSCCs as LDHA abrogation demonstrates no effect on tumor initiation or progression despite reduced glucose uptake and glycolytic intermediates [97]. However, they further demonstrate that *LDHA*-deficient tumors exhibited increased compensatory glutamine uptake, glutaminase activity, and reductive glutamine metabolism. This result demonstrates the flexibility by which SCCs maintain redox homeostasis and implicates the importance of maintaining intracellular antioxidant pools in SCCs. Glutamate and cysteine are essential precursors in de novo glutathione synthesis. ASCT2 (SLC1A5) mediates the influx of glutamine in exchange for cysteine efflux while cystine-glutamate antiporter xCT (SLC7A11) couples cystine import with glutamate export. Tumors may functionally couple these amino acid transporters to balance both intracellular cysteine and glutamate necessary for GSH synthesis (Figure 1) [98,99]. Suggestive of this molecular dependence is the observation that HNSCC and ESCCs are addicted to glutamine, the uptake of which is mediated by upregulated ASCT2 expression in HNSCC and necessary for GSH synthesis [100,101]. Furthermore, high ASCT2 expression is associated with decreased survival in both OSCC and HNSCC patients [101,102]. Work by Momcilovic et al. demonstrate that suppression of glycolysis by chronic mTOR inhibition elicits an adaptive upregulation of glutaminolysis via the GSK3 signaling axis in LSCC and HNSCCs, thereby not only defining a unifying metabolic signature of SCCs but also highlighting the metabolic plasticity by which SCCs sustain antioxidant anabolism [103].

### 3.3. Lactate and the Tumor Microenvironment

The metabolic reprogramming of the tumor microenvironment and the associated metabolic crosstalk between stroma and cancer cells also contributes to antioxidant defenses and tumor aggressiveness. Recent evidence demonstrates the cigarette smoke-induced metabolic reprogramming of cancer-associated fibroblasts (CAFs) towards a highly glycolytic phenotype. The highly glycolytic phenotype induces metabolic coupling and enhances the aggressiveness in HNSCCs, implicating oxidative stress in not only altering squamous metabolism but also shifting tumor stroma into a pro-oncogenic metabolic environment [104]. Additionally, CAFs may facilitate the glycolytic switch of HNSCCs by secreting HGF, which in turn induces bFGF secretion from HNSCCs to promote fibroblast proliferation [105]. Paracrine signaling and metabolic crosstalk within the tumor microenvironment results in concomitant increases in glycolysis in both tumor-associated stroma and tumor cells, thereby acidifying the extracellular space via monocarboxylate transporter-mediated lactate excretion [106]. This acidic milieu promotes immune evasion and drives tumor invasion especially at the tumor-stroma interface by increasing matrix metalloprotease (MMP) expression and rewiring of the transcriptome involved in the expression of an alternative splicing-dependent tumor invasion program [107,108]. Attenuated tumor EMT and invasion upon glycolytic inhibition in HNSCC in recent studies implicate glycolysis in facilitating metastasis [109,110].

Given the metabolic rewiring that SCCs undergo to redirect glucose flux into antioxidant generating pathways, lactate from the microenvironment and utilized as a respiratory substrate may serve to maintain energy homeostasis in response to decreased glucose flux into the TCA cycle. Faubert et al. demonstrate that NSCLCs, especially those with highest ^18^F-FDG uptake and papillary, solid, and squamous histology, preferentially uptake and utilize circulating lactate, not glucose, to fuel the TCA cycle as demonstrated by elevated lactate-to-3PG labeling ratios [111]. Identification of monocarboxylate transporters MCT1 and MCT4 as prognostically significant and generally highly expressed in many SCC subtypes further indicates the essential contribution of lactate transport in SCC oncogenicity [94,112,113,114,115,116]. Rather than primarily a waste product of glycolysis to be eliminated, lactate serves as a critical carbon shuttle and respiratory fuel enabling energetic resilience in metabolically fluctuating conditions and facilitating the diversion of glucose flux into essential anabolic pathways. Thus, the degree to which cancers redirect glucose utilization may determine the propensity by which lactate is catabolized.

Concomitant increases in MCT4, which exports lactate, and glycolysis in cigarette smoke-exposed CAFs and MCT1, which facilitates lactate uptake, and mitochondrial metabolism in HNSCCs demonstrates the essential metabolic coupling driving resistance to cell death and enhanced migration [104]. Furthermore, glucose deprivation in CESCCs, and the associated mitochondrial impairment and increase in ROS, resulted in MCT1 and CD147 upregulation and MCT1-CD147 heterocomplex accumulation, thereby increasing lactate uptake to combat oxidative stress and inducing migration toward more metabolically favorable environments in a lactate-independent manner [117,118]. Thus, MCT1 may not only permit SCCs to tolerate fluctuations in glucose availability by increasing lactate uptake but also dictate tumor invasiveness. However, the non-catabolic respiratory contribution of lactate to macromolecular biosynthetic pathways cannot be discounted, and lactate utilization may be dependent not only on alterations in glucose metabolism but also on oxygen status as lactate oxidation occurs in well-oxygenated regions such as lung tumors, which are well-perfused [114].

Rather than a universally one-dimensional idiosyncrasy of all cancers, the Warburg effect represents a complex, multi-faceted feature that cancers in different contexts utilize uniquely to fulfill various cellular needs, which may help explain the diversity in explanations for this phenomenon. In the SCC context, however, these data taken together reveal the remarkable coordination and adaptation in the uptake and utilization of glucose, glutamine, and lactate to fuel anabolic antioxidant generation while maintaining energy homeostasis, ultimately driving squamous-specific metabolic flexibility.

## 4. Interplay between Oncogenic Drivers and Metabolic Alteration

Comprehensive genomic analyses characterizing the mutational landscapes of SCCs reveal key commonalities in chromosomal aberrations and oncogenic events even across SCCs of distinct anatomical sites [11,12,14,15]. Surprising, however, is mounting evidence demonstrating a patchwork of somatic mutations traditionally associated with cancer already present within normal tissue, especially in the skin, lung, and esophagus which may contribute to the high degree of cellular heterogeneity observed in SCCs [73,119,120]. Targeted gene-sequencing analysis of both physiologically normal skin and normal esophageal epithelium revealed strong positive selection for canonical SCC driver mutations such as *NOTCH1*, which exhibits mutation prevalence as high as 20% and 12 to 80% in normal skin and esophagus, respectively [119,120]. Furthermore, copy number gains (CNGs) critically linked with SCC development such as the amplification of chromosome 3q, which contains *PIK3CA*, *TP63*, and *SOX2*, were detected within normal esophageal tissue, and 3q26 CNG was recently reported to be an early event in LSCC development cooperating with mutant p53 to drive progression [121]. Ultimately, the mutational landscape of normal tissue demonstrates not only clonal expansion in the context of normal cells harboring mutations conferring a selective advantage but also more nuance and complexity in factors contributing to cancer initiation and intratumoral heterogeneity. In essence, the high frequency of cancer-associated genomic alterations extant within normal tissue begs the question: what precisely drives SCC initiation or development?

One potential explanation may be that the accumulation of oncogenic mutations working in concert with each other more potently drives tumor formation than individual genomic aberrations. Given the recent appreciation for the role initiating truncal mutations play in selecting following mutations and driving branching evolution, highly prevalent mutations identified in normal epithelium may not be sufficient to initiate malignant tumors despite being able to confer a growth advantage or drive clonal expansion [122]. Despite possessing a mutational pattern characteristic of a tumor suppressor gene in head and neck, lung, skin, and esophageal SCCs and evidence demonstrating pro-oncogenic function in early oral tumorigenesis, *NOTCH1* mutations occur significantly more frequently in normal esophageal epithelium (30–80%) than in ESCC (~10%) [120,123,124]. In contrast, *TP53* mutations remain less common in normal esophageal tissue than in ESCCs, suggesting that order indeed matters in the context of SCC initiation and progression.

Although macroscopic clonal expansions were detected in many normal human tissues, tissues in which a majority of SCCs originate, the skin, esophagus, and the lung, experience the highest burden of somatic mutations and clonal expansions, suggesting that disparity indeed exists in the mutational landscape of normal tissues potentially due to differential environmental exposure, tissue architecture, microenvironmental cues or other extrinsic or intrinsic factors [73]. Despite the increased frequency of somatic mutations within SCC tissues of origin, however, distinct patterns of mutations emerge within somatic tissues such those involved in keratinocyte differentiation (*NOTCH1*, *NOTCH3*, *TP53*) and redox stress response (*NFE2L2*, *TP63*, *CUL3*), suggesting the existence of selective pressure driving the preferential survival or expansion of cells harboring advantage traits specific to SCCs. Striking commonalities in mutagenic and oncogenic mechanisms of disparate SCC-associated extrinsic risk factors may thereby potentially explain certain mutational patterns unique to SCCs.

**Figure 2:**
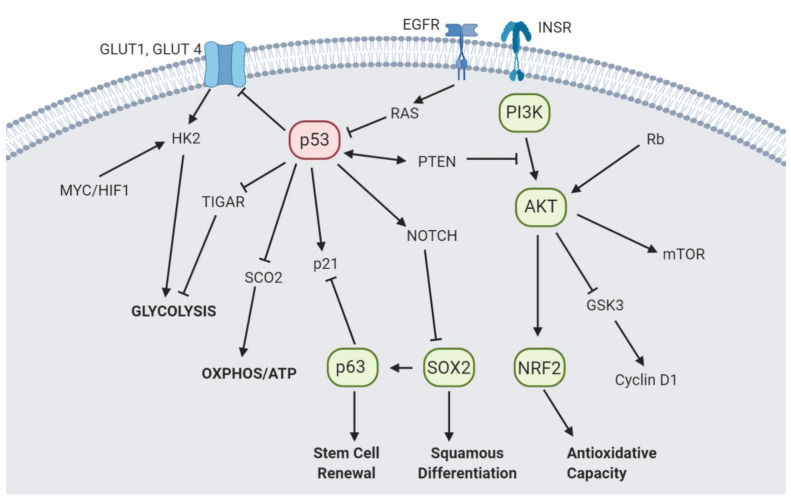
Overview of pathways frequently altered in SCCs demonstrating key metabolic and antioxidant functions. Proteins in green are highly expressed, and proteins in red are frequently inactivated.

Aberrant growth signaling alone, however, is not sufficient to drive proliferation but necessitates an essential coordination of metabolic processes and nutrient uptake to fulfill the bioenergetic and synthetic demands of uncontrolled growth [125]. Therefore, malignant transformation entails the alteration of metabolic pathways, which may be mediated by oncogenes traditionally thought to drive incessant proliferation and other hallmarks of cancer, and is far from a secondary consequence [126]. In essence, although the mutational signatures of SCCs and their functional roles in oncogenesis have been well discussed in the prevailing literature, evidence highlighting the critical interplay between oncogenic drivers and metabolic alteration uniquely pertaining to SCCs has only recently been coming to light (Figure 2).

### 4.1. p53

Tumor suppressor p53 sits at the nexus of many critical cellular processes including cell cycle regulation, genomic stability maintenance, apoptosis, and metabolism, thereby orchestrating a multifaceted response to diverse stresses. Mutations in tumor suppressor p53 represent the most frequent genetic aberrations across all SCCs and drives tumorigenesis by loss of wild-type p53 function, dominant-negative effects over the wild-type allele, or gain-of-function properties [127,128]. Wild-type p53 is generally thought to regulate energy production by suppressing glucose flux into glycolysis by inhibiting the expression of glucose transporters 1 and 4, transcriptionally inducing TIGAR, which redirects glucose flux into the PPP, and decreasing phosphoglycerate mutase (PGM) expression, among many other mechanisms of dampening glycolysis [129]. Concurrently, wild-type p53 maintains mitochondrial integrity and promotes oxidative phosphorylation via activation of synthesis of cytochrome *c* oxidase 2 (SCO2) and induction of glutaminase 2 (GLS2) expression, which increases glutamate and alpha-ketoglutarate production and consequently mitochondrial respiration [129]. Therefore, loss of p53 function can be thought of as generally tipping the metabolic balance towards glycolysis. Furthermore, p53 loss of function lifts p53-dependent repression on SLC7A11 leading to an increase in cystine uptake and resistance to ferroptosis, a ROS and iron-dependent form of cell death [130]. Beyond merely lifting restrictions on enhanced glycolytic flux, aberrations in p53 may function to actively drive the pro-proliferative Warburg effect and adapt to nutrient starvation through gain-of-function (GOF) effects. Hotspot mutants R175H, R248Q, and R273H p53 have been demonstrated to promote GLUT1 membrane translocation via RhoA-ROCK signaling activation in vitro and in vivo, thereby enhancing glucose influx and supporting anabolic growth [131]. However, not all p53 mutants are created equal. Recently, R248W but not R175H p53 was demonstrated to retain wild-type p53 ability of attenuating oxidative stress and promoting the switch to de novo serine biosynthesis in serine-limiting conditions [132]. Moreover, mutant p53 directly interacts with NRF2 to promote nuclear localization and binding to select antioxidant-response element (ARE) containing promoters, thereby driving a pro-survival oxidative response under excessive intracellular ROS [133]. Conversely, R175H and R280K mutant p53 has been demonstrated to increase ROS through NADPH oxidase 4 (NOX4) upregulation, which catalyzes superoxide production by transferring electrons from NADPH to oxygen [134]. Given the pro- and anti-oxidant roles of mutant p53, it may be possible that SCCs may either select for certain mutant p53 with antioxidant GOF effects or may necessitate the dual function of mutant p53 to maintain ROS below cytotoxic levels. Ultimately, however, the context-dependency of mutant p53 and whether certain mutants may be selected for have yet to be well-demonstrated in SCCs.

Despite the dearth in evidence of mutant p53 function in SCCs, studies have revealed that mutant p53 increases lipid production, aerobic glycolysis and invasion by preferentially binding AMPKα subunit in HNSCCs, leading to inhibition of AMPK activation, which normally functions to limit anabolic processes [135]. Furthermore, mutant p53-induced reduction in AMPK activation decreases AMPK-mediated FOXO3a phosphorylation, thereby de-repressing FOXM1 expression in HNSCCs [136]. In addition to transcriptionally regulating the cell cycle and maintaining genomic stability, FOXM1, upregulated early in HNSCC tumorigenesis, promotes the Warburg effect by transcriptional activation of GLUT1, HK2, and LDHA in multiple cancer cell lines and critically combats oxidative stress by inducing ROS scavenger gene expression [137,138,139,140]. Thus, mutant p53 may additionally exert pro-glycolytic effects via FOXM1 upregulation. While emerging evidence suggests GOF roles for p53, the contributions of mutant p53 alone or in cooperation with other oncogenic proteins to metabolic reprogramming have yet to be stratified by specific mutation or elucidated in the SCC context, which ultimately warrants further investigation especially given the high prevalence of p53 mutations in SCCs.

### 4.2. p63 and SOX2

p63 and SOX2 canonically function as key transcriptional regulators critical in maintaining stem cell pluripotency and cell fate determination including driving squamous epithelia differentiation [141,142]. Within SCCs even of diverse anatomical origins, ΔNp63 and SOX2 are highly expressed due to the frequent genomic amplification of the region between chromosome 3q26 and 3q28, suggesting the squamous lineage-specific oncogenic contributions of ΔNp63 and SOX2 [143]. The observation that ectopic SOX2 expression results in squamous lineage restriction in autochthonous mouse models of lung cancer further demonstrates the SCC-specific oncogenicity of SOX2 [144]. Amplified ΔNp63 via cooperation with other oncogenic events such as Ras and β-catenin activation or repression of tumor suppressors p53 and p73 have been well established in the prevailing literature to endow pro-proliferative effects and promote malignant progression in SCCs [145,146,147,148,149]. Furthermore, p63 and SOX2 have been demonstrated to jointly occupy multiple genomic loci in lung and esophageal SCCs, driving an oncogenic program distinct from maintaining pluripotency [150]. However, ΔNp63 and SOX2 not only drives aberrant growth via proliferative signaling in a SCC-specific context but also coordinates metabolic reprogramming to fuel anabolic growth and control oxidative stress. ΔNp63 alone directly induces HK2 expression to drive glucose metabolism and protection from oxidative stress within primary keratinocytes, and the ΔNp63-HK2 axis likely exists in SCCs as well to promote metabolic reprogramming [151]. Recently, Hsieh et al. revealed that ΔNp63 and SOX2 form a complex that binds to and transcriptionally activates an intronic enhancer cluster of *SLC2A1*, which encodes GLUT1, driving markedly increased glucose influx in lung, esophageal, and head and neck SCC cell lines [81]. The ΔNp63/SOX2-GLUT1 mediated glucose influx anabolically fuels GSH and NADPH synthesis, which is attenuated by p63 or SOX2 ablation. Critically, GLUT1 overexpression rescues the oxidative stress and cell death experienced by both SCC isogenic cell lines and xenograft tumors upon ΔNp63 depletion. Furthermore, Wang et al. demonstrate that ΔNp63 overexpression can transcriptionally activate of GSH redox pathways genes and upregulate glutathione metabolism in LSCCs, conferring survival against ROS-inducers and matrix detachment, in cooperation BCL-2 family proteins, and promoting metastasis [152]. Ultimately, these data evince the critical role ΔNp63 jointly with other key regulators plays in orchestrating the metabolic reprogramming via multiple mechanisms necessary, and given the frequent overexpression of ΔNp63, the management of oxidative stress may be a core phenotypic feature of SCCs driven by an SCC-specific transcriptional program. However, the metabolic functions of ΔNp63 and SOX2 are only recently emerging within SCCs, and further evidence elucidating their specific contributions in SCC initiation and development remain necessary. Given the essential roles both transcription factors play in normal squamous epithelial development and stem cell maintenance, it is likely that SCCs may hijack this mechanism, initially selected for etiological mechanisms of pathogenesis, to not only control ROS but also promote aberrant proliferation.

### 4.3. PI3K/AKT

Phosphatidylinositol 3-kinase (PI3K) is a critical downstream signaling component of the growth factor receptor tyrosine kinases (RTKs), and signaling through this pathway is also activated by various cytokines, growth factors, insulin, and other hormones. In its canonical role, PI3K phosphorylates and activates protein kinase B (AKT), which then catalyzes the phosphorylation of downstream targets to not only direct cellular growth, survival and redox homeostasis but also regulate the essential metabolic needs of cells [153]. Given its critical role in supporting growth, PI3K/AKT signaling is frequently altered in many cancers including colon, ovarian, lung, esophageal, head and neck, breast, and prostate cancer, among others, and drives oncogenesis through downstream effectors such FOXO, MYC, SREBP2 and NFR2 [154,155,156].

Dysregulation of PI3K/AKT has been linked with hyperactivation of Nrf2, a master transcriptional activator that induces expression of antioxidant proteins, including GSH, and regulates metabolic reprogramming in highly proliferative cells [157]. Nrf2 additionally promotes purine nucleotide synthesis and glutamine use in proliferating cells under the direction of PI3K/AKT [157]. Elevated GSH biosynthesis stimulated by oncogenic PI3K/AKT signaling drives resistance to oxidative stress and facilitates anchorage-independent growth and tumor spheroid initiation in PI3K-driven breast cancers [158]. Given the reliance of squamous cells on elevated antioxidative capacity and a high anabolic demand, PI3K emerges as an essential player in SCC malignancy by stimulating antioxidant generation in addition to its well-characterized role as a mitogenic signaling pathway. Genomic amplification of PIK3CA, which activates the p110α catalytic subunit of PI3K, is common in SCCs and leads to deregulated PI3K/AKT signaling [159]. Hsieh et al. and others demonstrated that aberrant PI3K/AKT signaling drives GLUT1 plasma membrane localization to enhance glucose influx which, in turn, fuels NADPH and GSH synthesis via the PPP and de novo serine biosynthesis pathway, respectively [81,160,161,162]. Upregulated PI3K/AKT signaling has also been demonstrated to enhance hexokinase 2 (HK2) activity, which in turn increases NADPH synthesis via the PPP [81,163]. Activating mutations in RTKs such as EGFR and HER2, which are frequently overexpressed in HNSCC and LSCC, also drive PI3K overexpression and oncogenic signaling via the mTOR pathway to coordinate the uptake and utilization of multiple nutrients, such as glucose, glutamine, nucleotides, and lipids [158,164]. Under the control of PI3K/AKT, mTORC1 alters glucose metabolism via a shift from OXPHOS to glycolysis, and upregulates HIF1a expression to radically increase nutrient uptake. SREBP upregulation by AKT-mediated mTORC1 activation also promotes the generation of NADPH and metabolites critical for growth and proliferation of SCC cells [165]. Given that PI3K pathway mutant breast cancers regress upon inhibition of GSH synthesis and cisplatin treatment in vivo, a similar metabolic vulnerability can be reasonably expected in SCC cancers and is seen in ESCCs [86,158].

Furthermore, elevated insulin signaling in SCCs drives a feedforward mechanism in which insulin activates the PI3K/AKT pathway, which in turn upregulates GLUT1 expression and glucose intake. The cells respond to hyperglycemia by rapidly increasing insulin signaling, promoting a cycle that overall meets anabolic demand and fuels enhanced proliferation [166,167]. Therefore, given the essential function PI3K/AKT signaling serves in linking proliferation, survival, and metabolism in SCCs, targeting insulin signaling may yield effective therapies tailored to the unique characteristics of SCC.

### 4.4. Nrf2

Nuclear factor erythroid 2-related factor 2 (Nrf2) is a master transcriptional regulator of enzymes involved in ROS detoxification and elimination, such as glutathione-*S*-transferase A2 (GSTA2) and NADPH:quinone oxidoreductase 1 (NQO1) and can be activated by multiple stimuli including increased ROS or electrophilic species or decreased reduced glutathione [168]. Under oxidatively challenging conditions, Nrf2 is stabilized by the inactivation of its E3 ligase Keap1 and translocates to the nucleus where it binds antioxidant response elements (AREs) to induce cytoprotective enzyme expression. In SCCs of diverse origins, activating mutations in *NFE2L2*, which encodes Nrf2, or loss-of-function in *KEAP1* remain frequent events, which leads to significantly elevated redox potential and confers resistance to radiation therapy [169,170]. That somatic Nrf2 mutations remain strikingly most frequent in SCCs even of disparate origin not only clearly implicates ROS in SCC development but also the indispensability of antioxidant upregulation to SCCs [171,172]. Nrf2 activation cooperates with other key oncogenic events to promote tumorigenesis and resistance to therapy. *Trp53* and *Keap1* loss have been demonstrated to not only promote airway basal stem cell expansion in in vivo murine models but also drive LSCC pathogenesis via Nrf2-mediated ROS reduction, inducing resistance to oxidative stress and ionizing radiation [169]. Interestingly, TGF-beta signaling-induced p21 expression stabilizes Nrf2 in SCC stem cell populations, resulting in tumor heterogeneity and chemotherapeutic resistance [173].

Nrf2 mounts an antioxidant defense by not only transcriptionally activating antioxidant enzymes but also metabolically rewiring cells to anabolically support enhanced antioxidant production [157,174,175]. Fu et al. reveal that hyperactive Nrf2 not only drives GSH metabolism but also upregulates glycolytic, PPP, and NADPH-synthesis genes in mouse esophagus, with Nrf2 ablation, PKM2 ablation, and glycolytic inhibitors suppressing human ESCC proliferation in vitro. Furthermore, Nrf2 has been demonstrated to directly promote the PPP, nucleotide synthesis, NADPH production, and glutamine metabolism, especially in conjunction with active PI3K/AKT signaling, which promotes the nuclear accumulation of Nrf2 [157]. Thus, proliferative signaling directly augments the metabolic reprogramming induced by constitutive Nrf2 stabilization, thereby revealing direct crosstalk and coordination between signaling and metabolism.

Given the robust etiological association of oxidative stress with SCCs and transformative contributions of ROS, aberrations in Nrf2 signaling may be an early event in squamous initiation and carcinogenesis initially combatting toxic oxidative stress but later serving to promote survival in highly proliferative cells. In support of this hypothesis, Rolfs et al. demonstrate that Nrf2 activation in precancerous mouse keratinocytes confers pro-tumorigenic properties and induces increases in GSH and purine metabolism pathways as well as enhancing the expression of enzymes involved in GSH and NADPH production [175]. Although pharmacological Nrf2 activation by sulforaphane treatment alone did not affect proliferation in both mouse and human keratinocytes, Nrf2 activation inhibited ROS-induced damage and apoptosis thereby enhancing survival. Furthermore, biopsies of actinic keratosis lesions, which frequently progress to SCC, were found to highly express Nrf2 targets NQO1 and SRXN1, thus suggesting the early involvement of Nrf2 activation in SCC initiation [176]. Therefore, Nrf2 may serve as a critical survival factor at multiple stages of tumorigenesis. Nrf2 initially promotes the survival and clonal expansion of precancerous cells, as demonstrated by Jeong et al., most able to activate Nrf2 upon ROS induction from a myriad of exogenous sources associated with SCC [169]. Upon acquisition of oncogenic drivers, Nrf2 serves to attenuate oncogene-induced oxidative stress. Furthermore, Nrf2-induced metabolic reprogramming supports proliferation in conjunction with oncogenic drivers by rewiring metabolic flux into anabolic pathways. Ultimately, constitutive Nrf2 expression via activating mutations in *NFE2L2* or Keap1 loss is a defining feature that almost all SCCs share that may present a targetable vulnerability to modulate the efficacy of current therapies.

## 5. Current Therapeutic Strategies

Prognoses for advanced SCCs, especially those occurring in the esophagus and the lung, remain among the poorest across all cancer types despite advances in targeted therapies and immune checkpoint inhibitors [33,177]. Neoadjuvant chemoradiation followed by surgical resection are standard treatment options for certain SCCs including locally advanced ESCC and LSCC, and postoperative chemoradiation has demonstrated improved control and disease-free survival in HNSCC, although adverse events are significantly increased [178,179,180]. Treatment of non-resectable or metastatic SCCs with cytotoxic chemotherapy cisplatin or carboplatin with 5-fluorouracil also remain standard supportive or palliative strategies [181,182,183,184]. Clinical trials investigating the efficacy of anti-EGFR therapies have generated mixed results in various SCCs. For instance, the monoclonal antibody cetuximab demonstrated minimal benefit in LSCC, moderate disease control in unresectable CSCC, increased locoregional control and reduced mortality of cetuximab in combination with radiation in HNSCC, and no efficacy in combination with chemotherapy in local and advanced ESCCs [16,185,186,187]. Anti-angiogenic agents also demonstrate limited efficacy in SCCs. Although VEGF inhibitor bevacizumab in combination with chemotherapy increased median progression-free survival in HNSCC and CESCC, overall survival was not affected, and life-threatening adverse events ultimately rendered bevacizumab treatment unacceptable for these indications [188,189,190]. Immune checkpoint inhibitors have been in evaluation in SCCs in recent years and demonstrate durable responses in certain patients. The anti-PD-1 antibody pembrolizumab has been approved by the FDA for the treatment of platinum-refractory HNSCC, recurrent or metastatic CSCC, advanced PD-L1-positive CESCC, and locally advanced or metastatic ESCC based on several KEYNOTE trials [191,192,193,194]. Moreover, pembrolizumab in combination with chemotherapy resulted in improved overall survival and prolonged disease-free progression in LSCC, leading to FDA approval for the treatment of metastatic LSCC [195]. However, despite remarkable improvements and durable responses observed in certain patient populations, overall response rates for immunotherapy, including SCCs as observed in the KEYNOTE trials, are limited with an estimated 12.46% of patients responsive to checkpoint inhibitors [196]. Given the varied and moderate efficacies of available treatments and the limited target population of immune checkpoint inhibitors, investigation into novel therapeutic strategies tailored to the unique characteristics of SCCs still remain essential. The metabolic dependencies in SCCs, and their intersections with oncogenic pathways, exposes a critical liability exploitable through therapies targeting metabolic-redox circuitry. Although the metabolic flexibilities of SCCs may limit the efficacy of monotherapeutic approaches, a combinatorial strategy of metabolic modulators and chemotherapy may not only prove to be effective through synergistic killing but also preclude and overcome drug resistance as metabolic reprogramming plays significant roles in chemoresistance [197,198]. Here we discuss potential metabolic strategies to potentiate the ROS-inducing effects of chemotherapy.

## 6. Targeting Squamous Cancers Metabolically

### 6.1. Targeting Glucose Reliance

Although targeting glucose metabolism has yielded varied and unsatisfactory outcomes due in part to varying glucose dependencies across cancers, narrow therapeutic indices, and tumor-intrinsic metabolic flexibility, SCCs nevertheless may be the cancer type most rationally targetable by glucose restriction [199]. The strict reliance of SCCs on glucose to maintain redox homeostasis renders such cancers vulnerable to perturbations in glucose uptake, and Goodwin et al. indeed demonstrate the in vivo anticancer effects of GLUT1 and glycolytic inhibition via WZB117 and 2-deoxy-D-glucose (2DG), respectively, in LSCCs [76]. 2DG was also demonstrated to selectively kill HNSCC cells in combination with cisplatin by precipitating oxidative stress [200]. Furthermore, Hsieh et al. reveal the preclinical efficacy of systemic glucose restriction via ketogenic diet or SGLT2 inhibition, which prevents renal glucose reabsorption, in potentiating platinum-based chemotherapies [81]. Decreasing blood glucose via systemic glucose restriction consequently reduces blood insulin, which thereby attenuates insulin-activated PI3K/AKT signaling. Recent evidence has demonstrated that ketogenic diet or SGLT2 inhibition enhances the efficacy of PI3K inhibitors by inhibiting the insulin feedback mechanism that normally maintains glucose homeostasis in model tumors in mice [201]. Given the frequent genomic amplification of PIK3CA and reliance on PI3K/AKT signaling observed in SCCs, systemic glucose restriction not only precipitates oxidative stress in tumors but also suppresses insulin-responsive growth pathways in a multi-pronged targeting of SCC oncogenicity: in essence, killing two birds with one stone [81]. Glucose restriction via ketogenic diet or SGLT2 inhibitors moreover presents a rapidly translatable adjuvant therapy potentiating the ROS-inducing effects of chemotherapies and radiation.

### 6.2. Vitamin C as a Prooxidant

Despite historical controversy over the efficacy of high dose vitamin C as an antineoplastic agent, new knowledge regarding its pharmacokinetics and recent clinical trials have sparked renewed interest in vitamin C as a viable therapy [202]. Vitamin C at micromolar physiological conditions acts as an antioxidant whereas at millimolar pharmacological conditions, vitamin C demonstrates pro-oxidant activity. Fully reduced vitamin C, ascorbate, is oxidized by free radicals or ROS extracellularly to dehydroascorbate (DHA), which is uptaken by GLUT1 and rapidly reduced back to ascorbate by reacting with GSH, thereby depleting the intracellular antioxidant pool. Furthermore, pharmacological ascorbate has been demonstrated to produce extracellular H_2_O_2_ to induce cell death [203,204]. High-dose vitamin C selectively kills KRAS-mutant colorectal cancer cells, which upregulate GLUT1, and recently a synergistic effect was demonstrated with fasting-mimicking diets in KRAS-mutated cancers [205,206]. Given that DHA is uptaken by GLUT1, it stands to reason that cancers highly expressing GLUT1 may be more susceptible to high-dose vitamin C treatment. Studies have indeed revealed that gastric cancer cells and VHL-defective renal cancer cells overexpressing GLUT1 are more susceptible to vitamin C treatment, and limited evidence suggests vitamin C also induces ROS-induced apoptosis in oral SCCs [207,208,209]. Additionally, high-dose vitamin C has been recently shown to enhance cancer immunotherapy in vivo [210]. Although clinical trials reveal no substantial benefit of vitamin C as a monotherapy and Hsieh et al. show that SCCs are in fact resistant to vitamin C presumably due to increased antioxidant capacity, ascorbate nonetheless may be effective in potentiating the ROS-inducing effects of traditional therapies [81,211]. Numerous clinical trials are currently investigating the efficacy of high-dose ascorbate in combination with radiation or chemotherapy in many cancers although none focus exclusively on SCCs [202]. Given the critical reliance antioxidant mechanisms and high expression of GLUT1, SCCs may be a cancer type uniquely vulnerable to combinatorial therapies utilizing vitamin C.

### 6.3. Targeting Nrf2

Frequent Nrf2 hyperactivation in SCCs either by *NFE2L2* activating mutations or *KEAP1* loss, which confers significant malignant potential and resistance to therapy, motivates the evaluation of strategies targeting the Nrf2 pathway. Various inhibitors of Nrf2 have been identified although none have yet to enter evaluation in clinical trials. The plant-derived quassinoid brusatol inhibits the Nrf2 pathway by enhancing ubiquitination and degradation independent of KEAP1 and sensitized an NSCLC cell line to cisplatin treatment [212]. The small molecule ML385, identified via high throughput screening, interacts with the Nrf2 DNA-binding domain to prevent binding to AREs and was found to enhance cytotoxicity of chemotherapeutic agents in NSCLC cells including an Nrf2-mutant LSCC cell line [213]. Halofuginone, another molecule identified via high-throughput screening, suppresses Nrf2 accumulation by repressing protein synthesis via prolyl-tRNA synthetase inhibition and ultimately sensitizing an ESCC cell line harboring mutant Nrf2 to chemotherapy [214]. Multiples lines of evidence demonstrating the in vitro ability of Nrf2 inhibition to sensitize SCCs to chemotherapy make Nrf2 an attractive target for evaluation in clinical trials.

### 6.4. Targeting Glutamine Metabolism

The striking glutamine addiction observed in some cancers, including SCCs, underscores the emergence of glutamine uptake and metabolism as attractive therapeutic targets in recent years [215]. Glutamine is a key intermediate in anabolic pathways, permits metabolic flexibility in lung SCCs upon glucose restriction, activates the MAPK pathway independent of Ras, serves as a mitochondrial substrate, and, once converted to glutamate, can be exchanged for cysteine to fuel GSH synthesis as well serve as a substrate itself [103,215,216]. Glutaminase 1 (GLS1) catalyzes the first step in glutamine metabolism in the conversion of glutamine to glutamate and ammonia and is frequently overexpressed in cancers. Currently, the GLS1 antagonist CB-839, is being evaluated in clinical trials as both monotherapy and in combination with chemotherapy and has demonstrated measured success in certain cancers [217,218,219,220]. CB-839 treatment has also been shown to overcome adaptive glutamine metabolism in lung SCC in vivo and also exhibits efficacy in ESCC cells resistance to CDK4/6 inhibition in in vitro and xenograft models [100,103]. Furthermore, ASCT2 silencing and inhibition via the novel small-molecule V-9302 have demonstrated in vivo efficacy in limiting xenograft tumor growth in HNSCCs by mTOR pathway suppression and oxidative stress induction [101,221]. The EGFR-inhibitor cetuximab additionally induces ASCT2 endocytosis and exhibits synergy with PDK1 inhibition by precipitating ROS, an effect rescued by N-acetyl cysteine treatment [222]. Therefore, these preclinical findings motivate the clinical evaluation of combinatorial strategies targeting glutamine metabolism in SCCs.

## 7. Concluding Remarks

SCCs in the past have suffered from a critical dearth of a unified understanding of their distinct tumorigenic properties due in part to high heterogeneity and myriad anatomical origins and only recently have etiological and molecular commonalities been coming to light. There is no doubt that identifying targetable vulnerabilities at the core of a cancer’s oncogenicity has yielded remarkable and leaping advances in therapy as with the identification of the BCR-ABL fusion protein in CML and the development of Gleevec. Although such “magic bullets” as first imagined by Paul Ehrlich may be far and few between, the characterization of the SCC landscape, far from a purely random and varied bag of genomic aberration, yields a common thread tied to squamous etiology and identity that may be targetable. Here, we posit a preliminary perspective that SCCs divided mostly by convenience via anatomical origin are in fact distinct from other cancers and consistent within in their genetic alterations, including determinants of squamous differentiation *TP63* and *SOX2* and the antioxidant response regulator Nrf2, that ultimately coordinate oncogenic and metabolic pathways to drive antioxidant generation. Although the nuances of each SCC in relation to their unique stromal environments as well as differing etiologies cannot be discounted, considering SCCs as a whole and investigating convergent oncogenic and metabolic pathways may yet uncover targetable liabilities at the nexus of SCCs.

## Figures and Tables

**Table 1:** Summary of epidemiological risk factors for SCCs, mechanism of action, and common genetic aberration associated with such.

Risk Factor	SCC Subtype	Mechanism of Action	Common Gene Mutations
Alcohol Consumption	HN, Esophageal	-Acetaldehyde-DNA adduct formation -Oxidative stress induced DNA damage -Lipid peroxidation products -Inhibition of DNA repair	*TP53*, *NOTCH1*, *CDKN2A*, *CCND1*, *PIK3CA*, *TAF1L*
Cigarette Smoking	HN, Lung, Esophageal	-Oxidative stress induced DNA damage -Mutations in guanine base pairs resulting in G/T substitutions	*TP53*, *TP63*, *EGFR*, *PIK3CA*
UV exposure, UV radiation (UVA, UVB),	Skin (cutaneous)	-DNA damage resulting in C/T substitutions -Production of reactive oxygen species (ROS). 8-oxo-dG formation; G/T transversions form during DNA replication.	*TP53*, *NOTCH1*, *2 CDKN2A*, *FAT1*, *CASP8*, *HRAS*, *AJUBA*, *KMT2D*
Infections EBV	HN	-Viral nuclear proteins EBNA2, EBNA3A, 3B, 3C activate MAPK/Jun, PI3K/AKT and B-catenin-signaling pathways	*TP53*, *CDKN2A*, *CCND1*
HPV	HN, Cervical	-E6 and E7 oncoproteins target p53 and pRb tumor suppressor functions	*TP53*, *PIK3CA*, *FBXW7*, *NOTCH2*, *RB*
